# Vitamin-D and associated biomarker profiles in patients with chronic heart failure

**DOI:** 10.12669/pjms.42.7.15018

**Published:** 2026-07

**Authors:** Fazil Choban Almammadov, Gulnara Alisha Jafarova

**Affiliations:** 1 Fazil Choban Almammadov, PhD Department of Family Medicine, Azerbaijan Medical University, Baku, Azerbaijan; 2 Gulnara Alisha Jafarova, PhD Department of Biochemistry, Azerbaijan Medical University, Baku, Azerbaijan

**Keywords:** Chronic heart failure, Vitamin-D deficiency, biomarkers, BNP, NT-proBNP, galectin-3, FGF-23, endothelin-1, VEGF-A

## Abstract

**Objectives:**

Vitamin-D deficiency is common in chronic heart failure (CHF) and may be linked to disturbances in mineral metabolism and cardiovascular biomarkers. This study investigated the association between Vitamin-D status and biochemical and cardiovascular markers across different CHF stages.

**Methodology:**

This cross-sectional observational study was conducted between 2023 to 2024 at three tertiary care centers in Baku, Azerbaijan (Republican Clinical Center, Sabunchu Clinical Center, and 1st Clinical Medical Center). The study included 219 patients with chronic heart failure (CHF), classified according to the New York Heart Association (NYHA) functional classification: 123 patients in class I–II and 96 patients in class III–IV. Serum Vitamin-D, mineral parameters, and cardiovascular biomarkers: BNP, NT-proBNP, galectin-3, parathormone (PTH), endothelin-1, fibroblast growth factor-23 (FGF-23), and vascular endothelial growth factor A (VEGF-A) were measured. Correlation analyses were performed to assess relationships between Vitamin-D levels and these markers.

**Results:**

Vitamin-D levels decreased with worsening NYHA class. Advanced CHF was associated with mineral metabolism disturbances and increased myocardial, neurohumoral, and vascular biomarkers. Significant inverse correlations were observed between Vitamin-D concentrations and BNP, NT-proBNP, galectin-3, and FGF-23, highlighting interconnected changes among these markers in CHF progression.

**Conclusion:**

Lower Vitamin-D levels are associated with greater functional severity of CHF and unfavorable biomarker profiles, suggesting that worsening Vitamin-D deficiency may be involved in disease progression and clinical deterioration.

## INTRODUCTION

Chronic heart failure (CHF) remains one of the most significant challenges in modern medicine.^1^ Despite advances in diagnostic, therapeutic, and preventive strategies, CHF is still associated with high mortality, frequent hospitalizations, and multiple comorbidities. Consequently, additional pathophysiological markers are sought to improve risk stratification and disease monitoring. Coronary artery disease (CAD), a major form of cardiovascular disease (CVD), is frequently associated with dyslipidemia and other modifiable risk factors, highlighting the significant burden of cardiovascular disorders in clinical populations.^2^

Vitamin-D deficiency has recently attracted considerable attention in cardiovascular research. It represents a widespread global health problem, affecting approximately 40.4% of the world’s population.^3^ Low Vitamin-D levels have been linked to CHF, AH, and atrial fibrillation (AF).^1^,^4^ Experimental and clinical data suggest that Vitamin-D deficiency is associated with activation of the renin–angiotensin–aldosterone system (RAAS), increased inflammatory activity, and endothelial dysfunction.^5^,^6^ Clinical evidence further indicates that Vitamin-D supplementation may improve physical performance in patients with heart failure, highlighting its potential therapeutic relevance.^7^

Vitamin-D plays a key role in calcium homeostasis and myocardial contractility. Reduced levels may impair calcium influx into cardiomyocytes, contributing to systolic dysfunction.^8^ Additionally, Vitamin-D deficiency is associated with secondary hyperparathyroidism, characterized by elevated parathyroid hormone (PTH) levels, which may promote myocardial fibrosis, left ventricular hypertrophy (LVH), and adverse cardiac remodeling.^9^ Experimental studies also indicate that calcitriol can inhibit cardiomyocyte hypertrophy and reduce natriuretic peptide secretion. Collectively, these findings suggest that Vitamin-D status reflects integrated metabolic and cardiovascular alterations in CHF.

Beyond clinical severity, Vitamin-D deficiency has been linked to changes in biomarkers involved in myocardial stress, fibrosis, inflammation, and vascular dysfunction. Galectin-3, a marker of myocardial fibrosis, increases with advancing CHF stages and correlates with low Vitamin-D levels.^10^ Natriuretic peptides, including B-type natriuretic peptide (BNP) and N-terminal pro-B-type natriuretic peptide (NT-proBNP), are established indicators of cardiac wall stress and disease severity, and have been reported to vary in relation to Vitamin-D status.^11^

Moreover, Vitamin-D deficiency has been associated with alterations in additional biomarkers such as fibroblast growth factor-23 (FGF-23), endothelin-1 (ET-1), and vascular endothelial growth factor (VEGF).^12^,^13^ FGF-23, a regulator of mineral metabolism, suppresses active Vitamin-D synthesis and is implicated in myocardial hypertrophy and adverse cardiovascular outcomes. Elevated ET-1 contributes to endothelial dysfunction and cardiac remodeling, while interactions between ET-1 and FGF-23 have been suggested in CHF progression.^14^,^15^ VEGF is involved in angiogenesis, collateral circulation, and prognosis in heart failure.^16^

Taken together, a comprehensive evaluation of Vitamin-D status alongside these biomarkers may provide additional insight into the pathophysiological processes accompanying CHF progression and improve disease assessment and monitoring. The aim of the study was to investigate the role of Vitamin-D deficiency in the pathophysiology of chronic heart failure.

## METHODOLOGY

This cross-sectional observational study enrolled patients sequentially during routine outpatient or inpatient visits at three tertiary care centers in Baku, Azerbaijan (Republican Clinical Center, Sabunchu Clinical Center, and 1st Clinical Medical Center) between 2023 to 2024. Seasonal variations in Vitamin-D levels were considered during recruitment. A formal sample size calculation was not performed; all eligible patients meeting the inclusion criteria during the study period were consecutively enrolled.

### Participants

A total of 219 CHF patients aged 30–89 years (mean 61.5 ± 0.7) were included, classified by New York Heart Association (NYHA) functional class: 123 as I–II (mean 60.4 ± 1.0) and 96 as III–IV (mean 62.9 ± 1.0). Patients were clinically stable, without recent hospitalization or acute decompensation. Primary etiologies included coronary artery disease (CAD), arterial hypertension, and dilated cardiomyopathy. Among all CHF patients, 198 (90.4%) had a history of CAD; patients with reduced ejection fraction (HFrEF, EF<40%) were included.

### Control group:

The control group consisted of 51 age- and sex-matched healthy volunteers aged 25–77 years (mean 54.3 ± 2.4), with serum Vitamin-D levels within the normal range, recruited from hospital staff and individuals undergoing routine health examinations at the same institutions during the same study period. Controls had no history of cardiovascular, renal, or acute inflammatory diseases.

### Exclusion criteria:

Exclusions included acute or chronic renal or hepatic failure, infectious or inflammatory diseases, recent diuretics or steroid use, Vitamin-D or calcium supplementation within two months, hypoparathyroidism, hypothyroidism, and eGFR <60 mL/min/1.73 m².

### Echocardiography:

Two-dimensional echocardiography with color Doppler and Simpson’s biplane method was performed by experienced cardiologists using GE Vivid E95 and Philips EPIQ 7 devices. EF values (median, Q1–Q3) were: NYHA I–II: 68.0% (62–71%), NYHA III–IV: 40.0% (33–46%), controls: 37.5% (31.5–41.5%).

### Blood collection and biomarker analysis:

Fasting blood samples were collected by venipuncture in the morning. Serum Vitamin-D [25(OH)D], BNP, and NT-proBNP were analyzed immediately; other biochemical parameters were stored at −70 °C until analysis. Assays included: creatinine, calcium, phosphorus, magnesium (commercial kits), BNP/NT-proBNP and PTH (electrochemiluminescence), Vitamin-D (ELISA), VEGF-A and endothelin-1 (ELISA). Intra- and inter-assay CVs were<10%. Vitamin-D deficiency: <20 ng/mL; insufficiency: 20–30 ng/ml; sufficiency: >30 ng/ml.

### Statistical Analysis:

Data are presented as median (Me), 25th (Q1), and 75th (Q3) percentiles. Mann–Whitney U and Kruskal–Wallis tests were used for group comparisons; fold changes were calculated as median ratios. Spearman correlation evaluated relationships between variables. Significance was set at p<0.05. SPSS version 26 was used.

### Ethical Approval:

The study complied with the Helsinki Declaration and was approved by the Ethics Committee of Azerbaijan Medical University (approval No. 3/2018, September 16, 2018).

## RESULTS

No statistically significant difference in serum creatinine was observed among the patients. Vitamin-D deficiency worsened progressively with CHF severity: levels were 52.1% lower in NYHA I–II (p<0.001) and 2.1-fold lower in NYHA III–IV (p<0.001) compared to controls, with class III–IV showing 65.7% lower levels than I–II (p<0.001) (Table-I).

**Table I:** Changes in functional and biochemical parameters of patients with chronic heart failure according to the stage of the disease.

Parameters	Groups											
	Control				NYHA class I-II				NYHA class III-IV			
	*M±S.D.*	*Me*	*Q1*	*Q3*	*M±S.D.*	*Me*	*Q1*	*Q3*	*M±S.D.*	*Me*	*Q1*	*Q3*
Creatinine, mg/dl	0.86±0.2	0.88	0.76	0.96	0.90±0.17	0.92	0.79	1.00	0.89±0.18	0.89	0.76	1.01
p p_1_					0,112				0,488 0,376			
p_H_	0,283											
Vitamin-D, nq/ml	43.5±8.8	42.0	38.0	48.0	28.6±9.0	29.0	21.0	37.0	20.5±11.5	17.5	12.5	28.0
p p_1_					<0,001				<0,001 <0,001			
p_H_	<0,001											
PTH, pq/ml	38.4±13.8	37.7	25.9	50.9	58.6±12.9	57.1	48.3	67.2	61.9±14.1	61.2	50.6	69.4
p p_1_					<0,001				<0,001 0,099			
p_H_	<0,001											
P, mmol/l	1.14±0.14	1.16	1.05	1.23	1.89±0.46	1.84	1.50	2.31	1.91±0.42	1.88	1.62	2.22
p p_1_					<0,001				<0,001 0,804			
p_H_	<0,001											
Ca, mq/dl	9.3±0.5	9.4	8.9	9.6	9.2±1.0	9.1	8.4	9.8	8.6±0.8	8.6	8.0	9.1
p p_1_					0,083				<0,001 <0,001			
p_H_	<0,001											
Mg, mmol/l	0.95±0.1	0.97	0.90	1.00	0.87±0.07	0.88	0.80	0.93	0.82±0.16	0.81	0.78	0.86
p p_1_					<0,001				<0,001 <0,001			
p_H_	<0,001											
EF (ejection fraction)	66.9±5.2	68.0	62.0	71.0	39.5±7.4	40.0	33.0	46.0	36.8±7.5	37.5	31.5	41.5
p p_1_					<0,001				<0,001 0,003			
p_H_	<0,001											
BNP, pq/ml	46,2±3.3	50,0	29,0	63,0	541,1±138.6	538,0	423,0	628,0	663,7±215.0	634,0	557,5	759,5
p p_1_					<0,001				<0,001 <0,001			
p_H_	<0,001											
NT-pro-BNP, pq/ml	72,5±34.4	65,0	46,0	96,0	1270,1±429.2	1285,0	954,0	1485,0	1662,0±431.2	1688,5	1556,5	1845,5
p p_1_					<0,001				<0,001 <0,001			
p_H_	<0,001											
Galectin-3, nq/ml	7,3±2.6	7,0	5,5	9,6	14,2±2.7	14,4	11,8	16,3	18,1±4.9	18,1	15,4	19,4
p p_1_					<0,001				<0,001 <0,001			
p_H_	<0,001											
FGF-23, pq/ml	48.3±13.0	48.2	39.1	61.2	55.0±10.1	53.4	48.9	62.9	58.2±13.4	56.4	47.1	65.9
p p_1_					0,003				<0,001 <0,001			
p_H_	<0,001											
VEGF-A, pq/ml	35.3±8.3	36.4	28.7	43.1	155.2±36.0	154.4	124.0	186.5	300.5±73.8	312.7	274.0	347.4
p p_1_					<0,001				<0,001 <0,001			
p_H_	<0,001											
Endothelin-1, pq/ml	5.6±1.5	5.9	4.0	6.7	9.2±2.0	9.1	7.9	10.5	11.8±2.7	11.5	9.9	13.6
p p_1_					<0,001				<0,001 <0,001			
p_H_	<0,001											

***Note:*** *M* represents the mean value; *Me* – the median; *Q1* – the first quartile (25th percentile); *Q3* – the third quartile (75th percentile). *p* indicates statistical significance compared with the control group, and *p1* – compared with patients in NYHA class I–II. All p-values were calculated using the Mann–Whitney U test. p_H_ - the overall comparisons across NYHA functional classes were assessed using the Kruskal–Wallis test.

Calcium decreased by 9.3% in NYHA III–IV (p<0.001), while phosphorus increased by 58.6% in early stages and 62.0% in later stages (p<0.001). In class III–IV, calcium and magnesium decreased by 5.8% and 8.6% (p<0.001), respectively, with no significant change in phosphorus (p=0.804). PTH levels rose by 51.4% in NYHA I–II and 62.3% in III–IV compared to controls (p<0.001), with a 7.2% higher concentration in III–IV vs. I–II (p=0.099).

Ejection fraction (EF) was reduced in CHF patients: 39.5 ± 0.7% in NYHA I–II and 35.8 ± 0.8% in III–IV, versus 66.9 ± 0.8% in controls. EF decreased by 69.4% in I–II and 86.9% in III–IV (p<0.001), with III–IV being 10.3% lower than I–II (p=0.003).

BNP and NT-proBNP were markedly elevated: BNP increased 10.8-fold in I–II and 12.7-fold in III–IV (p<0.001), while NT-proBNP increased 19.8-fold and 26.0-fold, respectively (p<0.001). Increments from I–II to III–IV were 17.8% for BNP and 31.4% for NT-proBNP (p<0.001). Galectin-3 increased 2.0-fold in I–II and 2.6-fold in III–IV (p<0.001), with a 25.7% rise between functional classes (p<0.001).

FGF-23 rose 10.8% in I–II and 20.0% in III–IV (p=0.003 and p<0.001), reflecting myocardial remodeling linked to Vitamin-D deficiency and phosphate overload. VEGF-A increased 4.2-fold in I–II and 8.6-fold in III–IV (p<0.001), while endothelin-1 increased 54.2% and 95%, respectively, with III–IV being 26% higher than I–II (p<0.001).

Correlation analyses indicated negative associations between Vitamin-D and PTH (ρ= –0.179; p=0.008), galectin-3 (ρ=–0.275; p<0.001), VEGF-A (ρ=–0.289; p<0.001), and endothelin-1 (ρ=–0.288; p<0.001), and positive correlations with calcium (ρ= 0.241; p = 0.001) and magnesium (ρ= 0.143; p=0.035). NT-proBNP showed an inverse correlation with Vitamin-D (ρ = –0.136; p = 0.044). FGF-23 correlated positively with BNP (ρ= 0.381; p<0.001), NT-proBNP (ρ= 0.403; p<0.001), and VEGF-A (ρ=0.596; p<0.001). EF negatively correlated with VEGF-A (ρ= –0.154; p=0.023) and endothelin-1 (ρ= –0.265; p<0.001).

Overall, these findings highlight progressive Vitamin-D deficiency in CHF, accompanied by alterations in mineral metabolism, myocardial stress, fibrosis, and neurohumoral and vascular biomarkers.

## DISCUSSION

The present study demonstrates that Vitamin-D deficiency in patients with chronic heart failure (CHF) becomes progressively more pronounced with advancing NYHA functional class, suggesting its involvement in broader disturbances of bone-mineral metabolism, myocardial structure, intracellular signaling, and immune regulation.^17^ The active form of Vitamin-D, 1,25(OH)_2_D_3_, exerts its biological effects via the Vitamin-D receptor (VDR), contributing to the regulation of calcium–phosphate homeostasis, suppression of RAAS activity, reduction of oxidative stress, and modulation of inflammatory cytokine expression.^18^

In our cohort, early-stage CHF was characterized by reduced calcium and elevated phosphorus levels, whereas patients in NYHA class III–IV demonstrated combined reductions in calcium and magnesium alongside significantly increased FGF-23 levels.^19^ These changes reflect disturbances in mineral metabolism, including hypocalcemia, hypomagnesemia, and hyperphosphatemia, which may contribute to myocardial dysfunction and structural remodeling. In addition, markedly elevated PTH levels in advanced CHF are consistent with secondary hyperparathyroidism secondary to Vitamin-D deficiency. Elevated PTH may further impair myocardial contractility, enhance oxidative stress, and activate hypertrophic signaling pathways.^7^,^17^ These findings are consistent with observations reported in populations with a high prevalence of Vitamin-D deficiency, including South Asian cohorts.

Beyond mineral metabolism, Vitamin-D deficiency appears to be associated with activation of multiple neurohormonal, inflammatory, endothelial, and fibrotic pathways implicated in CHF progression. Reduced Vitamin-D availability may contribute to RAAS overactivation, macrophage-mediated inflammatory responses, and galectin-3–associated myocardial fibrosis.^10^,^20^ Endothelial dysfunction may be further exacerbated through increased endothelin-1 activity, reduced nitric oxide bioavailability, and dysregulated VEGF-A expression, collectively promoting adverse myocardial remodeling, interstitial fibrosis, and microcirculatory impairment.^21^–^23^

Although correlations between Vitamin-D levels and natriuretic peptides (BNP and NT-proBNP) were modest, they were statistically significant and consistent with previous studies reporting higher peptide concentrations and reduced cardiac function in patients with low 25(OH)D levels.^13^,^24^ Elevated BNP (>400 ng/l) has also been identified as an independent predictor of all-cause mortality and rehospitalization in CHF.^3^ Collectively, these findings suggest that vitamin-D status reflects an integrated pathophysiological milieu in CHF, linking disturbances in mineral metabolism with myocardial stress, fibrosis, and neurohumoral activation.

From a clinical perspective, biomarker assessment, including vitamin-D status, may provide additional value in routine heart failure management by supporting risk stratification and monitoring of disease severity.

### Limitations and future directions:

It includes a modest sample size and a cross-sectional observational design, which precludes causal inference. In addition, the study was conducted in a single-country, regional population, which may limit the generalizability of the findings to other populations. Therefore, prospective and interventional studies, particularly randomized controlled trials, are warranted to evaluate whether correction of Vitamin-D deficiency may improve biomarker profiles and clinical outcomes. Overall, our findings support the interpretation of Vitamin-D deficiency as a marker of disease severity and progression in CHF rather than a primary causal factor in its pathogenesis.

## CONCLUSIONS

The progressive decline in Vitamin-D levels with increasing NYHA functional class is closely associated with dysregulation of mineral metabolism, hormonal balance, and endothelial function, reflected by elevated FGF-23, VEGF-A, and endothelin-1 levels. These findings suggest that Vitamin-D status is closely linked to the complex pathophysiological processes underlying chronic heart failure, including cardiovascular remodeling, inflammatory activation, and immune dysfunction. Assessment of Vitamin-D status alongside these biomarkers may provide additional value for clinical monitoring and risk assessment in chronic heart failure.
